# The comparison of an accessible C-shaped partial stapled hemorrhoidopexy (C-PSH) versus circular stapled hemorrhoidopexy (CSH) in patients with grade IV hemorrhoids: a retrospective cohort study

**DOI:** 10.1186/s12876-023-03062-1

**Published:** 2023-12-16

**Authors:** Jun He, Meng-Dan Zhou, Wen-Jing Wu, Zhi-Yong Liu, Dong Wang, Guan-Gen Yang, Qin-Yan Yang, Zhong Shen

**Affiliations:** 1 Department of Colorectal Surgery, Hangzhou Third People’s Hospital, Hangzhou, 310009 Zhejiang People’s Republic of China; 2 Department of Nursing, Hangzhou First People’s Hospital, Hangzhou, 310006 Zhejiang People’s Republic of China

**Keywords:** C-shaped partial stapled hemorrhoidopexy, Partial stapled hemorrhoidopexy, Circular stapled hemorrhoidopexy, Grade IV hemorrhoids

## Abstract

**Objectives:**

The objectives of this study were to present an accessible C-shaped partial stapled hemorrhoidopexy (C-PSH) in the treatment of grade IV hemorrhoids and to assess long-term outcomes of this technique compared with circular stapled hemorrhoidopexy (CSH).

**Methods:**

Conventional CSH kits combined with an intestinal spatula were used for performing C-PSH. A total of 256 patients with grade IV hemorrhoids referred to Hangzhou Third People's Hospital between January 2016 and June 2017 were obtained: 122 (47.7%) with C-PSH, and 134 (52.3%) with CSH. After propensity score matching, 222 patients (111 in C-PSH group and 111 in CSH group) were ultimately analyzed. The primary outcome was the five-year recurrence rate of hemorrhoids. Secondary outcomes included intraoperative outcomes, postoperative outcomes and complications.

**Results:**

The operative time in the C-PSH group was slightly longer than that in the CSH group (*p* < *0.01*). The vertical length of rectal mucosa specimen in the C-PSH group was shorter than that in the CSH group (*p* < *0.01*). Compared with the CSH group, fecal urgency incidence and numeric rating scale (NRS) score at first defecation were lower in the C-PSH group (*p* < *0.05*). Major complication rate in the CSH group was higher than that in the C-PSH group (*p* = *0.03*). Five-year recurrence rate between the C-PSH group and CSH group was comparable (*p* > *0.05*). Multivariate Cox regression analysis revealed that constipation was an independent prognostic factor for hemorrhoidal recurrence.

**Conclusions:**

The accessible C-PSH seems to be a safe and effective technique in managing grade IV hemorrhoids. It has advantages in alleviating postoperative pain at first defecation, fecal urgency and major complications compared with CSH. It could be an alternative technique in the treatment of grade IV hemorrhoids.

## Introduction

Hemorrhoids are common benign diseases in colorectal surgery. It is difficult to determine its exact incidence in common population. A study of screening colorectal cancer in normal population reported that the prevalence of hemorrhoids approximated 39% and half of the participants were asymptomatic [1]. Hemorrhoids are usually classified into two categories: internal hemorrhoids and external hemorrhoids. The typical symptoms of hemorrhoids are bright red bleeding, tissue prolapse, anal pain, soiling or itching and so on. According to the extent of prolapse, internal hemorrhoids are graded from I to IV and the treatments differ with each grade [2, 3]. About ten to twenty percent of patients with symptomatic hemorrhoids require surgical treatment [4]. Circular stapled hemorrhoidopexy (CSH) is one of the effective techniques to treat prolapsed hemorrhoids [5–7]. Compared with conventional hemorrhoidectomy, the CSH is associated with advantages such as less pain and short recovery time [6, 8]. With the popularization of this procedure, however, many unpleasant feelings or complications such as fecal urgency, rectal stenosis and massive bleeding pointed to this technique have been recorded [5, 9, 10]. In order to decrease those weaknesses, an accessible C-shaped partial stapled hemorrhoidopexy (C-PSH) which based on CSH has been practiced in our team in recent years. The purposes of this study were to present the C-PSH technique in the treatment of grade IV hemorrhoids and to compare long-term outcomes of this technique with CSH.

## Patients and methods

### Patients

Consecutive patients, diagnosed with grade IV hemorrhoids, consented circular stapled hemorrhoidopexy or C-shaped partial stapled hemorrhoidopexy in the Department of Colorectal Surgery of Hangzhou Third People’s Hospital between January 2016 and June 2017 were obtained from electronic medical record system. The patients were informed the details of the two techniques and had the privilege of choosing one. Patients with severe diseases (cardiovascular diseases or cerebrovascular diseases), abnormal coagulation, colorectal diseases (thrombosis hemorrhoids, anal fistula, abscess, stenosis, inflammatory bowel disease and tumor), and age less than 18 years were excluded. The baseline characteristics including gender, age, body mass index (BMI), self-reported hemorrhoids duration and defecation situations (chronic diarrhea or constipation) were collected. Chronic diarrhoea was diagnosed when patient met the following criteria: stool consistency between types 5 and 7 on the Bristol stool chart and duration greater than 4 weeks [11]. The diagnosis of chronic constipation was referred to the functional constipation of Roma IV criteria [12].

### Surgical procedures

**Fig. 1:**
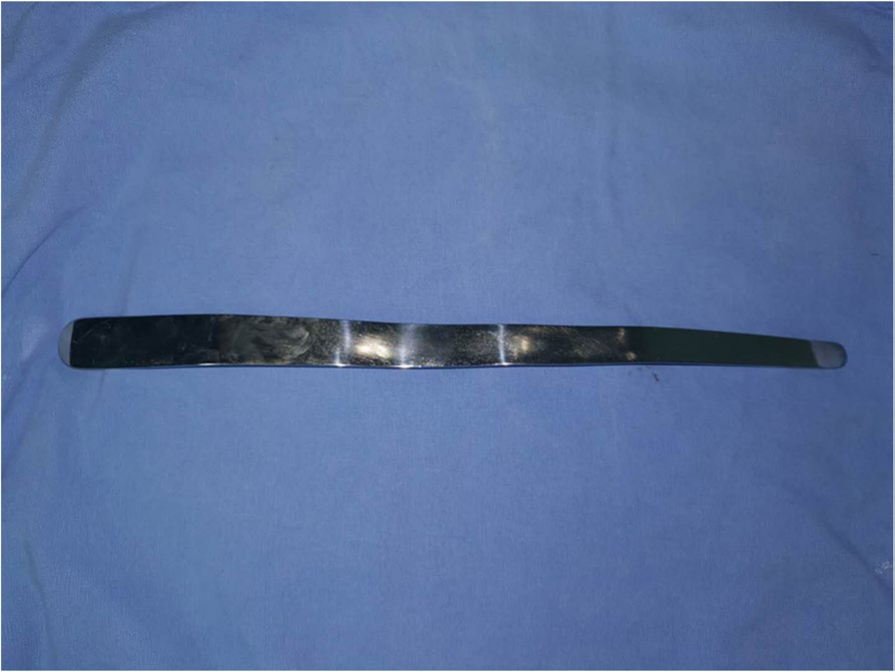
An intestinal spatula used for performing C-shaped partial stapled hemorrhoidopexy

**Fig. 2:**
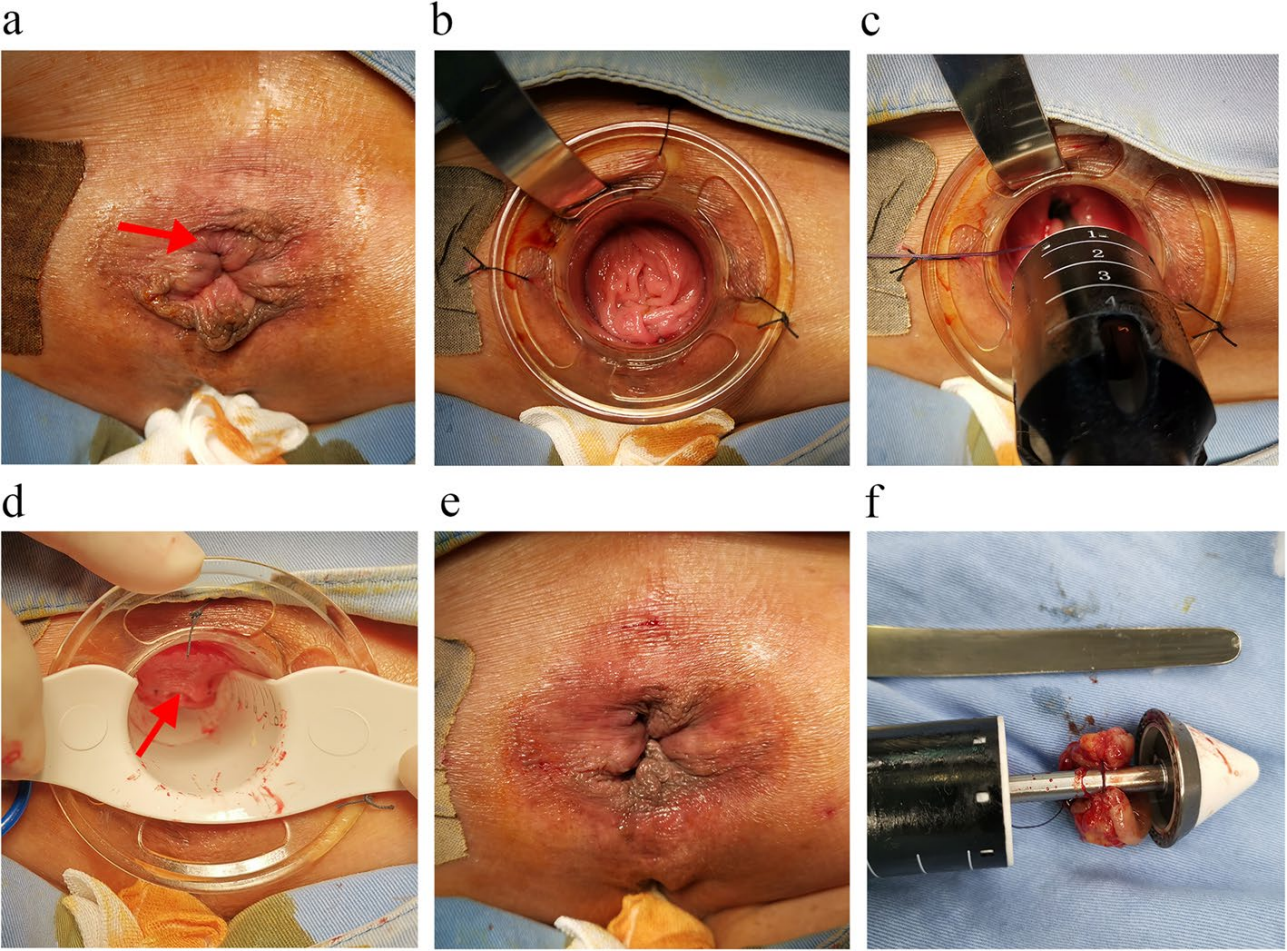
Procedure of C-shaped partial stapled hemorrhoidopexy. **a** A hemorrhoidal space was selected as an insertion point of intestinal spatula (red arrow); **b** The intestinal spatula was placed through the gap between anal canal and anoscope; **c** A C-shaped mucosal flap was secured to the rod; **d** A normal mucosal bridge between “dog ears” was preserved after the C-shaped partial stapled hemorrhoidopexy (red arrow); **e** Perianal appearance after C-shaped partial stapled hemorrhoidopexy; **f** The resected C-shaped specimen

All patients had a routine bowel-cleansing enema before surgery. After spinal anesthesia, the patients were placed in the prone Jack-knife position. Both the C-PSH and CSH procedures were conducted by the same group of doctors, all of whom possess over 25 instances of experience in performing C-PSH and CSH procedures. In the C-PSH group, a disposable stapler kit (PSHS34, Victor, China) combined with an accessible intestinal spatula (Fig. 1) were used for performing the C-PSH. After a routine anal dilatation, anal physical examination was performed to confirm hemorrhoidal positions and spaces between hemorrhoids (Fig. 2a). An anoscope was inserted through the anal canal and fixed. Then, a hemorrhoidal space was selected as an insertion point, and the intestinal spatula was carefully placed through the gap between the anal canal and anoscope (Fig. 2b). The top of the intestinal spatula reached 5 cm above the dentate line. A pursestring was placed approximately 3 cm above the dentate line by a 2–0 suture (Vicryl Plus, ethicon, USA), and the depth of suture within submucosa. Because of the intestinal spatula, the shape of pursestring was similar to alphabet “C”. The anvil of stapler was introduced and positioned above the C-shaped pursestring, and the suture was then secured to the rod (Fig. 2c). C-shaped mucosal flap was pulled into the barrel of the stapler, and the stapler was fired. A vaginal examination was routinely performed before stapler was fired in sexually active women patients. After removing stapler and intestinal spatula, a stapler bridge connecting the mucosectomies was formed and normal mucosal bridge was under it. The stapler bridge was then separated, “dog ears” were ligated by 3–0 suture (Vicryl Plus, ethicon, USA) (Fig. 2d). A routine inspection of anastomotic stoma was performed and anastomotic bleeding was controlled by using “8” suture. Perianal appearance after C-shaped partial stapled hemorrhoidopexy was shown in Fig. 2e. Finally, the residual skin tags were removed routinely after C-PSH procedure. The resected C-shaped specimen was shown in Fig. 2f. In the CSH group, the disposable hemorrhoids stapler kit (PSHS34, Victor, China) was also used for performing stapled hemorrhoidopexy as the method described by Longo [7]. After CSH procedure, the remaining external piles were also routinely excised. Postoperative managements including nursing care, dietary adjustments, and sitz baths, and postoperative analgesia were standardized.

### End points

The primary outcome was the five-year recurrence rate of hemorrhoids. Secondary outcomes included intraoperative outcomes, postoperative outcomes and complications. Intraoperative outcomes including operation duration, blood loss, vertical length of resected mucosa specimen and skin tags resection were collected. Operation time was recorded from disinfection to dressing. Blood loss was estimated by the same specification gauze (each gauze was represented as 5 ml). Specimens were routinely examined by the same group of pathologists. Postoperative outcomes included postoperative pain, fecal urgency and hospital stay. Postoperative pain was assessed by NRS (numeric rating scale, scores from 0 to 10; 0 as painless and 10 as worst pain) [13]. NRS scores were recorded at 1^st^ day, 2^nd^ day, 3^rd^ day, 7^th^ day and first defecation after surgery. Fecal urgency was defined as unable to defer defecation for more than 15 min [5]. Complications such as urine retention, haemorrhage, perianal abscess, rectal or vaginal fistula, rectal stenosis, and chronic anal pain were recorded.

The follow-up lasted until September 2022. In the first year, patients were routinely assessed by experienced colorectal surgeons at 1 week, 1 month, 3 months, 6 months and 1 year after surgery. Then, the follow-up was performed once a year. The follow-up visits included outpatient visit and telephone interview. During outpatient visits or telephone interviews, patients were queried about symptoms of hemorrhoids, such as painless bright red bleeding, prolapsing hemorrhoids, soiling, pain, itching, or any combination of these symptoms. Unless the patient was completely asymptomatic, they would be scheduled for a follow-up examination in the outpatient clinic. Any discomfort not limited to chronic anal pain, rectal stenosis and recurrence was recorded and received appropriate treatment. Recurrence is defined as at least one symptom recrudesce after 2 months symptom-free period and confirmed by an experienced colorectal surgeon [14].

### Statistical analysis

Statistical analysis was performed on SPSS (IBM, version 25.0, USA) or R software (R Core Team, version 3.5.2, Austria). The quantitative data were represented as mean (standard deviation) or median (range). The two-sample t-test or Mann–Whitney U test was used for comparing quantitative variables. The Chi-squared test or Fisher exact test was used for qualitative variables. The propensity to undergo C-PSH versus CSH was estimated by using a logistic regression model based on age, gender, body mass index, hemorrhoids duration, chronic diarrhea, and chronic constipation. The matching algorithm was the 1:1 nearest neighbor matching method. Propensity score matching (PSM) analysis was performed by using the matching package in R software. Recurrence related data was analyzed by log-rank test or multivariate Cox regression analysis. *P* < 0.05 was regarded statistically significant.

## Results

### Patients

**Fig. 3:**
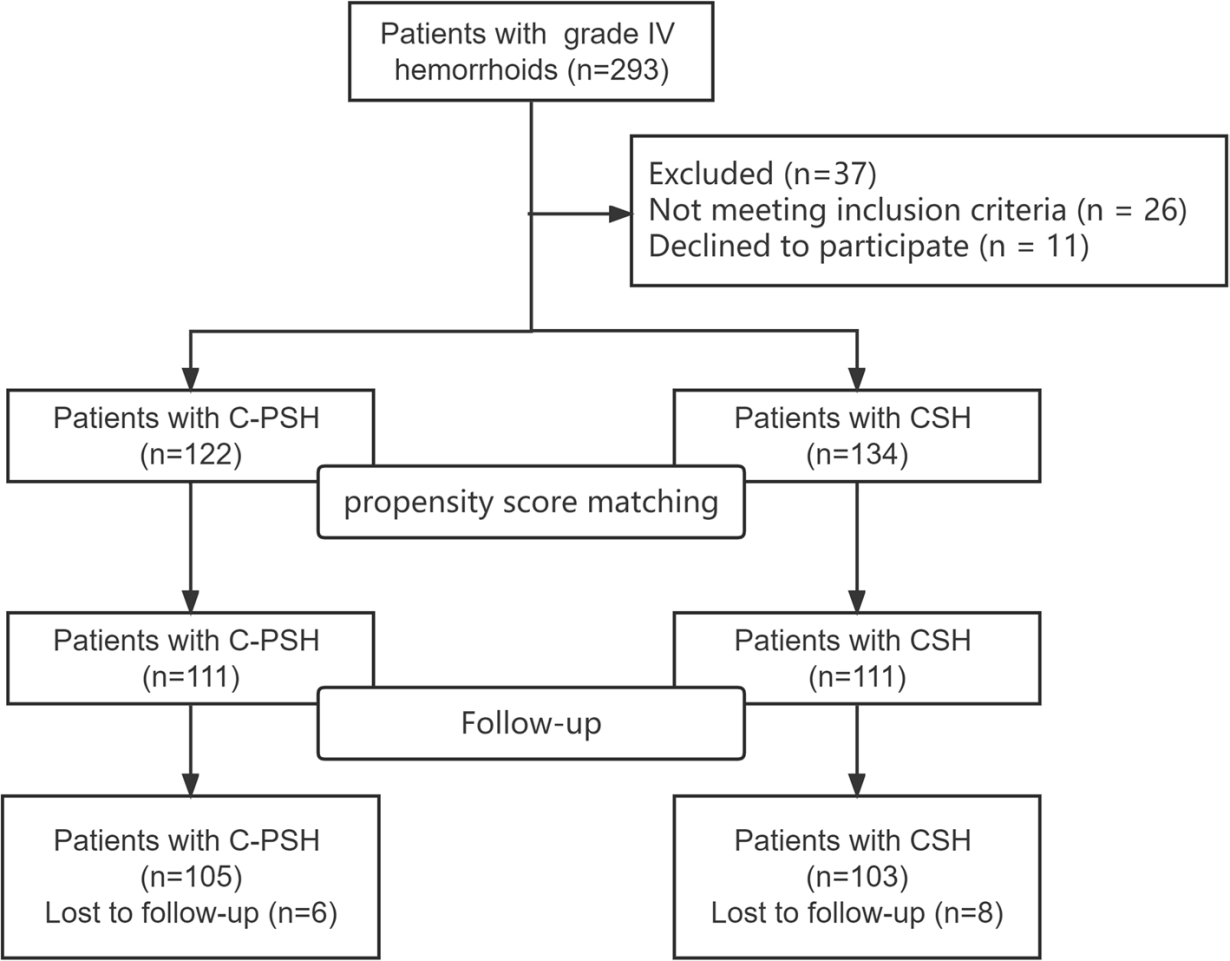
Patient flowchart. (C-PSH, C shaped partial stapled hemorrhoidopexy; CSH, Circular stapled hemorrhoidopexy)

A total of 256 patients met the inclusion criteria in the present study. Among the 256 patients, 122 (47.7%) underwent C-PSH, and 134 (52.3%) underwent CSH. After propensity score matching, a total of 222 patients were ultimately enrolled, with 111 in the C-PSH group and 111 in the CSH group (Fig. 3). A good balance was achieved, and the clinical features were shown in Table 1.

**Table 1:** Participant characteristics

Parameter	Unmatched Cohort		*P-value*	Matched Cohort		*P*-value
	CSH (134)	C-PSH (122)		CSH (111)	C-PSH (111)	
Gender			0.02*			*0.42*
Female	64 (47.8%)	76 (62.3%)		61 (55.0%)	67 (60.4%)	
Male	70 (52.2%)	46 (37.7%)		50 (45.0%)	44 (39.6%)	
Age (years)	52.5 (27.0–79.0)	51.0 (25.0–81.0)	*0.46*	54.0 (27.0–79.0)	50.0 (25.0–81.0)	*0.86*
BMI (kg/m^2^)	21.8 (2.0)	22.5 (1.8)	< 0.01*	22.1 (2.0)	22.5 (1.8)	*0.10*
Hemorrhoids duration (years)	8.0 (1.0–32.0)	7.0 (1.0–23.0)	0.02*	8.0 (1.0–25.0)	7.0 (1.0–23.0)	*0.45*
Chronic diarrhea			*0.38*			*0.67*
No	123 (91.8%)	108 (88.5%)		100 (90.1%)	98 (88.3%)	
Yes	11 (8.2%)	14 (11.5%)		11 (9.9%)	13 (11.7%)	
Chronic constipation			*0.18*			*0.73*
No	103 (76.9%)	102 (83.6%)		90 (81.1%)	92 (82.9%)	
Yes	31 (23.1%)	20 (16.4%)		21 (18.9%)	19 (17.1%)	

*Abbreviations*: *C-PSH* C-shaped partial stapled hemorrhoidopexy, *CSH* Circular stapled hemorrhoidopexy, *BMI* body mass index. * indicates significant difference

### Intraoperative and postoperative outcomes

The operation time in the C-PSH group was slightly longer than that in the CSH group (C-PSH vs. CSH, median 23 min vs. 21 min, *p* < *0.01*). The vertical length of rectal mucosa specimen was shorter in the C-PSH group (C-PSH vs. CSH, 3.11 ± 0.58 cm vs. 3.42 ± 0.43 cm, *p* < *0.01*). There were no significant differences in estimated blood loss, skin tags resection, urine retention, and hospital stay between the two groups (*p* > *0.05*) (Table 2).

**Table 2:** Comparison of intraoperative, postoperative and complication outcomes between the CSH and C-PSH groups after propensity score matching

Parameter	CSH (111)	C-PSH (111)	*P-value*
Operation time (minutes)	21 (13–56)	23 (15–52)	< 0.01*
Blood loss (ml)	10 (5–60)	10 (5–55)	*0.07*
Vertical length of rectal mucosa specimen (cm)	3.42 (0.43)	3.11 (0.58)	< 0.01*
Skin tags resection			*0.27*
No	74 (66.7%)	66 (59.5%)	
Yes	37 (33.3%)	45 (40.5%)	
Hospital stay (days)	5 (3–12)	5 (3–11)	*0.32*
Fecal urgency			0.03*
No	76 (68.5%)	90 (81.1%)	
Yes	35 (31.5%)	21 (18.9%)	
Urine retention			*0.72*
No	92 (82.9%)	94 (84.7%)	
Yes	19 (17.1%)	17 (15.3%)	
Major complication	9 (8.1%)	2 (1.8%)	0.03*
Massive bleeding	3 (2.7%)	1 (0.9%)	*0.61*
Rectovaginal fistula	0	0	*NA*
Anal stenosis	4 (3.6%)	0 (0%)	*0.13*
Rectal abscess	0	0	*NA*
Chronic anal pain	2 (1.8%)	1 (0.9%)	*1.00*

*Abbreviations*: *C-PSH* C-shaped partial stapled hemorrhoidopexy, *CSH* Circular stapled hemorrhoidopexy. * indicates significant difference

**Fig. 4:**
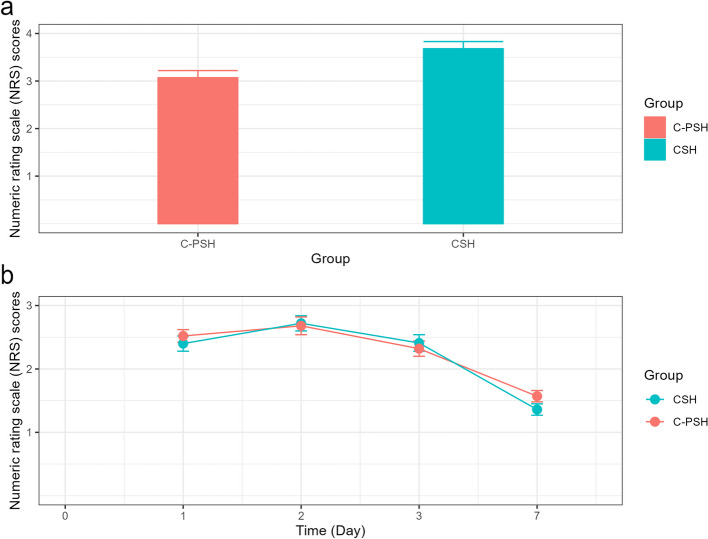
The numeric rating scale (NRS) scores after surgery. **a** NRS scores at first defecation. **b** NRS scores in 1^st^, 2^nd^, 3^rd^ and 7.^th^ postoperative days. (C-PSH, C shaped partial stapled hemorrhoidopexy; CSH, Circular stapled hemorrhoidopexy)

The fecal urgency incidence in the C-PSH group was lower than that in the CSH group (C-PSH vs. CSH, 18.9% vs. 31.5%, *p* = *0.03*) (Table 2). Compared with the CSH group, the NRS score in the C-PSH was lower than that in the CSH group at first defecation after surgery (C-PSH vs. CSH, 3.07 ± 1.60 vs. 3.68 ± 1.56, *p* < *0.01*) (Fig. 4a). However, the pain scores in 1^st^, 2^nd^, 3^rd^ and 7^th^ postoperative days between the two groups were comparable (all *p* > *0.05*) (Fig. 4b)

### Followed-up results

**Fig. 5:**
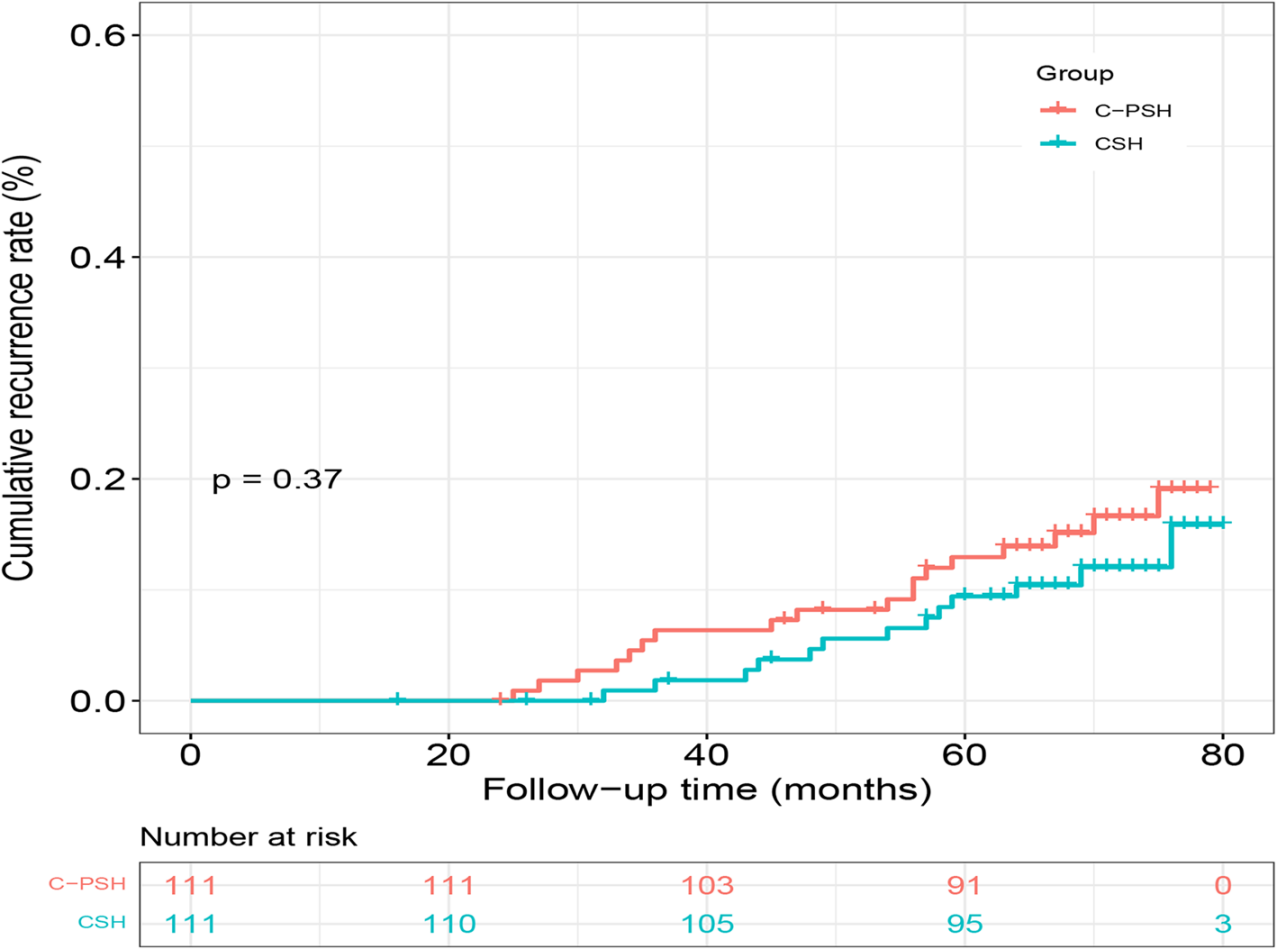
The comparison of cumulative recurrent rates between the C-PSH group and CSH group. (C-PSH, C shaped partial stapled hemorrhoidopexy; CSH, Circular stapled hemorrhoidopexy)

The median follow-up of this study were 70 months (24–79 months) in the C-PSH group and 68 months (16–80 months) in the CSH group. There were 14 (6.3%) patients lost to follow-up: 6 (5.4%) in the C-PSH group and 8 (7.2%) in the CSH group. One elder patient in the CSH group died from a severe pneumonia at 16 months after surgery, and the other patients dropped out of the study or lost contact. A total of 31 patients were confirmed hemorrhoidal recurrence: 18 (16.2%) in C-PSH group and 13 (11.7%) in CSH group. The five-year recurrence rates between the C-PSH group and CSH group were comparable (12.9% vs. 9.4%, hazard ratio = 1.43, *p* = *0.38*). There was also no significant difference in the cumulative recurrence rate between the two groups (C-PSH vs. CSH, 19.9% vs. 14.7%, hazard ratio = 1.39, *p* = *0.37*, Fig. 5). Among the recurrence patients, two patients with CSH and two cases with C-PSH underwent conventional hemorrhoidectomy due to significant prolapse and bleeding, and one patient with CSH due to pronounced bleeding and mild prolapse received rubber band ligation. The remaining patients were relieved by conservative treatments.

### Risk factors associated with hemorrhoidal recurrence

The results showed that constipation was an independent prognostic factor (hazard ratio, 4.48, 95%CI: 1.99–10.05, *p* < *0.01*). The operative modality was not a risk factor that contributed to the hemorrhoidal recurrence (hazard ratio, 1.60, 95% CI: 0.76–3.37, *p* = *0.22*) (Table 3).

**Table 3:** Multivariate Cox regression analysis of risk factors for hemorrhoidal recurrence in the matched cohort

Parameter	Hazard ratio (95% CI)	*P-value*
Gender (male vs. female)	1.48 (0.71–3.09)	*0.29*
Age (years)	1.01 (0.98–1.04)	*0.74*
BMI (kg/m^2^)	0.98 (0.81–1.20)	*0.88*
Hemorrhoids duration (years)	1.00 (0.93–1.08)	*0.96*
Chronic diarrhea (yes vs. no)	2.43 (0.84–7.04)	*0.10*
Chronic constipation (yes vs. no)	4.48 (1.99–10.05)	< 0.01*
Vertical length of rectal mucosa specimen (cm)	1.05 (0.54–2.03)	*0.88*
Skin tags resection (yes vs. no)	1.29 (0.61–2.71)	*0.50*
Treatment modality (C-PSH vs. CSH)	1.60 (0.76–3.37)	*0.22*
Major complication (yes vs. no)	1.43 (0.30–6.73)	*0.65*

*Abbreviations*: *CI* Confidence interval, *C-PSH* C-shaped partial stapled hemorrhoidopexy, *CSH* Circular stapled hemorrhoidopexy, *BMI* body mass index. Note: * indicates significant difference

### Complications

Major postoperative complication rate in the C-PSH group was significantly lower than that in the CSH group, (C-PSH vs. CSH, 1.8% vs. 8.1%, *p* = *0.03*) (Table 2). Nine patients suffered postoperative complications in the CSH group: 3 (2.7%) with massive hemorrhage, 4 (3.6%) with rectostenosis, and 2 (1.8%) with chronic anal pain. Two patients experienced postoperative complications in the C-PSH group: 1 (0.9%) with massive hemorrhage and 1 (0.9%) with chronic anal pain. Three patients in the CSH group with major postoperative complications required surgical treatments: two patients with rectostenosis and one patient with massive hemorrhage. One patient with C-PSH group suffered massive hemorrhage and was treated with surgery. The other patients were relieved by conservative treatments.

## Discussion

In this study, the long-term results showed that the hemorrhoidal recurrence rates between the C-PSH group and CSH group were comparable, and the constipation was an independent prognostic factor for recurrence. The C-PSH group had advantages in reducing fecal urgency, pain of first defecation after surgery, and major complications. However, compared with CSH group, slightly longer operation time and shorter vertical length of rectal mucosa specimen were observed in the C-PSH group.

The treatment strategies of hemorrhoids include medical and surgical treatments. It is assumed that surgical treatment is the most effective strategy for recurrent, or symptomatic hemorrhoids [2]. However, conventional surgery (e.g., Milligan-Morgan hemorrhoidectomy) has some disadvantages such as severe postoperative pain and prolonged convalescence [15]. And since anal cushion theory was proposed by Thomson, the treatment of hemorrhoids has been largely changed [16]. In 1998, Longo first reported the procedure of circular stapled hemorrhoidopexy (CSH), using a circular suturing device, to manage the hemorrhoidal disease by reduction of mucosal and hemorrhoidal prolapse [7]. Compared with conventional surgery, enhanced postoperative recovery and decreased postoperative pain were observed by using CSH. Since then, CSH has been spread widely. However, many side effects such as fecal urgency, anal stenosis, massive bleeding and other complications have been reported [5, 9, 10, 17]. In recent years, partial stapled hemorrhoidopexy (PSH), which is characterized by a special designed anoscope, has been introduced in clinical treatment, and the decreased drawbacks of stapled hemorrhoidopexy are reported [5, 14, 18]. By using this technique, partial rectal mucosa above the dentate line is resected and the mucosal bridges between the mucosectomies are reserved [5, 19]. Compared with CSH, the incidence of complications is largely reduced and long-term outcomes are comparable [14, 18, 19]. However, the devices of PSH, especially the specially designed anoscope, are not available in some areas. As a result, the popularization of this technique is largely restricted. Therefore, we presented a simplified C-PSH technique which utilized easily accessible instruments (intestinal spatula or tongue spatula) to preserve the rectal mucosa bridge during performing stapled hemorrhoidopexy.

According to our study, the procedure time of C-PSH was slightly longer than that of the CSH group. It may be attributed to the placement of intestinal spatula and ligation of “dog ears”. Although the vertical length of rectal mucosa specimen was longer in the CSH group, multivariate Cox regression analysis revealed that it was not an independent prognostic factor for hemorrhoidal recurrence. The postoperative fecal urgency was reported as high as 40% after CSH [20]. In this study, the incidence of urgency in the C-PSH group (18.9%) was much lower than that in the CSH group (31.5%). The reason for fecal urgency is not clear. It was speculated that foreign bodies and inflammation at the staple ring might cause such discomfort [18]. The reduced incidence in the C-PSH group may be interpreted by the reduction of staples residual and inflammatory response in the staple ring. Postoperative pain is usually inevitable in hemorrhoidectomy. Compared with conventional hemorrhoidectomy, the postoperative pain is largely reduced in stapled hemorrhoidopexy [8]. It should be noted that Chinese surgeons preferred to excise the residual skin tags after stapled hemorrhoidopexy. The reasons are as follows: skin tags are usually observed after CSH or C-PSH, and the aesthetic requirements of patients are considered. Besides, studies demonstrated that postoperative pain between patients with or without skin tags excision was similar [5, 21]. In the present study, the postoperative pains in the C-PSH group and CSH group were observed at a low level. And the first defecation pain in the C-PSH group was observed lower than that of CSH group. The reserved rectal compliance in C-PSH group might be the contribution.

Morbidity is one of the efficient indicators for assessing the safety of a technique. In this study, major complication rate in the C-PSH group was observed lower than that of the CSH group. Three patients in the CSH group and one patient with an insufficient mucosa bridge in the C-PSH group occurred massive bleeding. We postulated anastomotic stoma suffered excessive tension in defecation was a main reason. Rectostenosis is one of the common postoperative complications in stapled hemorrhoidopexy and usually occurs within four months after surgery [22]. No patient suffered stenosis in the C-PSH group, while 4 patients underwent this complication in the CSH group during the follow-up. The result is in accordance with previous study [5, 14]. It was supposed that excessive annular fibrosis around the staples might be the reason for stenosis. Due to the reserved mucosal bridge, the compliance of rectum is remained and rectal stenosis incidence in the C-PSH technique is largely decreased. Chronic anal pain was observed in three patients: two patients in the CSH group and one patient in the C-PSH group. All three patients were observed retained staples. The unpleasant feeling was relieved by removing the bare staples. It is postulated that persisting inflammation and excessive fibrosis are the causes [18, 23, 24].

Although the five-year recurrence rate (12.9%) and cumulative recurrence rate (19.9%) in the C-PSH group were higher than that in the CSH group, the difference was not significant. The recurrence rate of this study was lower than that reported by previous study. Tjandra et.al reported that the recurrence rate of hemorrhoidal disease after CSH was 25% [6]. Constipation has been regarded as a risk factor in hemorrhoidal development [2, 25]. Multivariate Cox regression analysis revealed that constipation was an independent prognostic factor for hemorrhoidal recurrence. Hence, management of constipation would be an effective means to reduce the probability of hemorrhoidal recurrence.

Although C-PSH has many superiorities in treatment of hemorrhoids, the drawbacks should not be neglected. One of the disadvantages is that majority of staples are retained after stapled hemorrhoidopexy, and it may cause metal artifacts in the magnetic resonance inspection [24]. And the general trauma is more severe than other treatments such as rubber band ligation. Hence, many surgeons are apt to adopt other techniques to relieve hemorrhoidal disease. Being aware of the potential weaknesses, the stapled hemorrhoidopexy was restricted to the patients with grade IV hemorrhoids in our team. Based on our C-PSH practices, we also want to share some preliminary experiences with operators. Firstly, the insertion point of spatula is not constant. Surgeons could select a hemorrhoidal space as the insertion point. Secondly, lubrication of spatula with paroline may make the insertion easily. Thirdly, insertion procedure should be performed carefully and slowly to avoid anal fissure or mucosal injury.

Our study has some limitations. First, all cases were from local Chinese patients, and whether C-PSH is superior to CSH surgery needs further validation in other populations. Additionally, there is a lack of control over lifestyle changes (such as dietary habits and bowel habits) that could potentially influence long-term surgical outcomes. Furthermore, this study serves as a preliminary exploration of the C-PSH technique and aims to introduce its accessibility. It is a single-center study with a small sample size and retrospective design. As a non-randomized controlled trial, this approach may introduce biases that could compromise the quality of our findings. While we employed Propensity Score Matching (PSM) to mitigate confounding biases, it cannot fully replace a randomized controlled trial. In the near future, it is essential to conduct multicenter, large-scale, randomized, and long-term studies to provide more robust evidence.

## Conclusions

In general, C-PSH seems to be a safety and efficacy technique for the treatment of grade IV hemorrhoids. By using this technique, the postoperative pain at first defecation, fecal urgency and major complications are observed to be decreased, and long-term recurrence rate is not significantly increased. The outcomes reveal that our simplified C-PSH could be an alternative technique in the management of grade IV hemorrhoids.

## Data Availability

The datasets generated and analyzed during the current study are available from the corresponding author on reasonable request.
